# Racial/Ethnic Inequities in Polysubstance Use Among Online Help–Seeking Sexual and Gender Minoritized People in San Francisco From 2022 to 2025: Cross-Sectional Study

**DOI:** 10.2196/82313

**Published:** 2025-09-12

**Authors:** Jarett Maycott, Sean Arayasirikul

**Affiliations:** 1 Center for Public Health Research San Francisco Department of Public Health San Francisco, CA United States; 2 The Legacy Center Joe C. Wen School of Population & Public Health University of California, Irvine Irvine, CA United States

**Keywords:** online samples, digital health, polysubstance use, sexual and gender minoritized community health, social media

## Abstract

This research letter characterizes racial/ethnic disparities in polysubstance use among 409 online help–seeking sexual and gender minoritized people in San Francisco. Findings demonstrate the central role of tobacco as a co-occurring substance for participants who are Black, Indigenous, and people of color compared to their White counterparts.

## Introduction

Polysubstance use, the concurrent or sequential use of more than 1 substance within a defined interval, is associated with multiple elevated health risks (eg, overdose, poor mental health, HIV) and disproportionately impacts sexual and gender minoritized (SGM) subgroups, such as transgender people and cisgender lesbian women [1-3]. Few studies have documented racial/ethnic disparities in polysubstance use among SGM populations [4]. Analyses of the 2021 National Survey on Drug Use and Health identified greater risk of polysubstance use among nonheterosexual respondents, illustrating SGM health disparities in substance use [5], yet little is known about how these disparities emerge in the critical moments before seeking help, particularly among SGM individuals using online platforms, where opportunities for digital heath intervention may be most immediate and impactful to populations least likely to have access to supportive public health services [5].

## Methods

### Study Design and Participants

We recruited 409 participants between 2022 and 2025 through Facebook, Instagram, and Grindr advertisements for individuals seeking help for substance use or treatment services and related topics (eg, HIV, mental health). Participants were eligible if they were men who have sex with men or transgender and gender expansive, were aged ≥18 years, were San Francisco residents, and had a mobile phone.

### Measures

We measured the following sociodemographic characteristics: age, race/ethnicity, gender identity, sexual orientation, HIV serostatus, housing, and income. Race/ethnicity was recoded into a dichotomous variable (1 for Black, Indigenous, and people of color [BIPOC] and 0 for White). We measured substance use by asking participants how many days in the last 30 they used tobacco, vape devices, and alcohol; engaged in hazardous drinking; and used marijuana, prescription opioids (eg, pain relievers), nonprescription opioids (eg, heroin, fentanyl), other prescription drugs (used nonmedically), other illicit substances (eg, crack, cocaine, amphetamines, methamphetamine, hallucinogens, or inhalants such as poppers), and any injected drugs. For each substance, we created a dichotomous variable for any reported use at least once in the prior 30 days (yes/no). We measured past-30-day polysubstance use by assessing the prevalent combinations of pairs of substances used in the last 30 days.

### Data Analysis

A correlation matrix heat map was used to illustrate the proportion of participants reporting each polysubstance use combination. We then constructed logistic regression models to examine associations between BIPOC identity and substance use pairs in the top quantile of prevalence (above 10% of the sample), adjusting for age, gender, sexual orientation, HIV status and housing stability, and socioeconomic status. Analyses were performed using R (version 2024.12.0). No missing data were recorded.

### Ethical Considerations

The study protocol was approved by the University of California, San Francisco institutional review board (20-33169). Participants provided signed informed consent and were given a US$30 gift card for completing the assessment. Data were deidentified to protect participants’ privacy and confidentiality.

## Results

**Figure 1:**
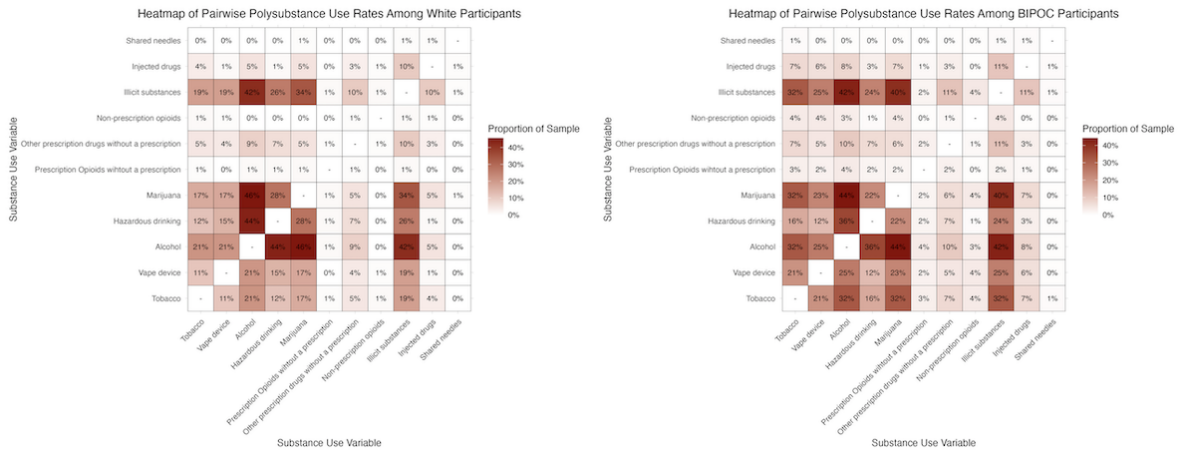
Heat map comparison of pairwise polysubstance use rates among White participants and Black, Indigenous, and people of color (BIPOC) participants.

**Figure 2:**
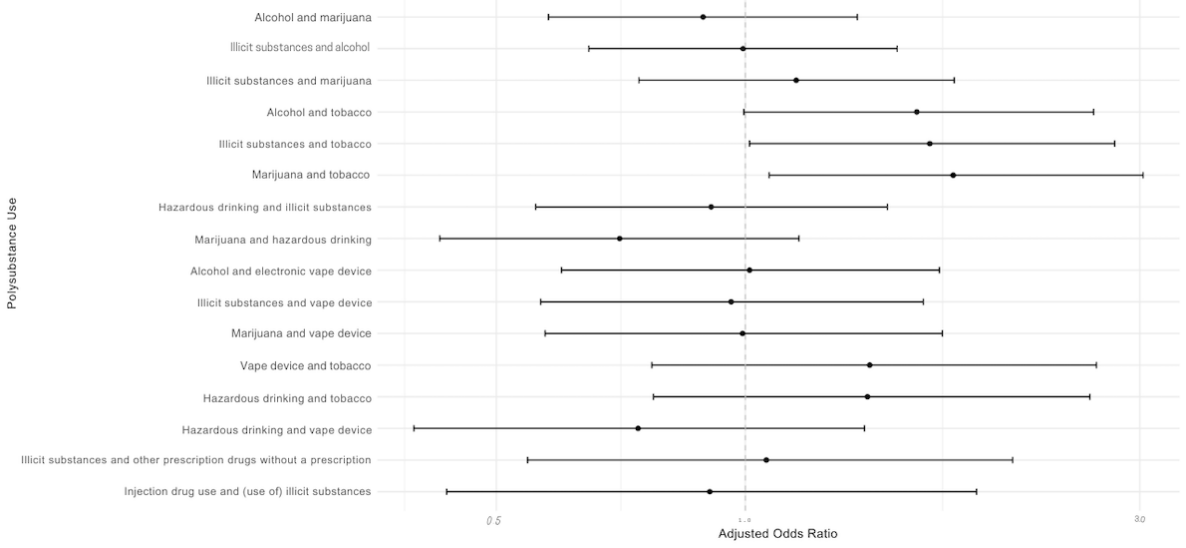
Likelihood of polysubstance use among sexual and gender minoritized participants who were Black, Indigenous, or people of color compared to sexual and gender minoritized participants who were White (in San Francisco from 2022 to 2025; N=409).

BIPOC participants comprised 58.7% (240/409) of the sample, with the largest group being Latine (113/409, 27.6%), followed by Asian and Pacific Islander and Native Hawaiian (50/409, 12.2%), multiracial/other (39/409, 9.5%), and Black/African American (38/409, 9.3%). Figure 1 presents 2 matrices, stratified by BIPOC identity, for White participants and BIPOC participants. Tobacco appears more frequently as a co-occurring substance across all substance use behaviors among BIPOC participants compared to White participants. Use of a vape product was also notably higher as a co-occurring substance in all categories for BIPOC participants. In contrast, hazardous drinking was less prevalent among BIPOC participants (86/240, 35.8%) than among White participants (74/169, 43.8%). Regression models (Figure 2) identified 3 substance use combinations with higher odds of use among BIPOC participants compared to White participants: alcohol and tobacco (adjusted odds ratio [aOR] 1.61, 95% CI 1.00-2.64), illicit substances and tobacco (aOR 1.67, 95% CI 1.07-2.80), and marijuana and tobacco (aOR 1.78, 95% CI 1.07-3.03).

## Discussion

We found that 3 combinations of polysubstance use involving tobacco—alcohol and tobacco, illicit substances and tobacco, and marijuana and tobacco—were more common among BIPOC participants than White participants. This pattern mirrors other research documenting higher tobacco use prevalence in BIPOC communities despite trending decreases in use nationwide [6]. This study has limited generalizability due to its cross-sectional study design, self-report bias, and its focus on online help–seeking individuals in San Francisco. Despite this, our results highlight a persistent gap in understanding how race intersects with tobacco use and its role in polysubstance use, particularly within minoritized populations actively seeking support online. Future research examining ecological dimensions of polysubstance use would further our understanding of the social context of polysubstance use.

## Abbreviations

aOR: adjusted odds ratio
BIPOC: Black, Indigenous, people of color
SGM: sexual and gender minoritized

## Data Availability

The datasets generated or analyzed during this study are not publicly available as they contain details that could be used to identify participants but are available from the corresponding author on reasonable request.
